# S80. NEUROCOGNITIVE FUNCTIONING IN YOUTH AT RISK OF SERIOUS MENTAL ILLNESS

**DOI:** 10.1093/schbul/sby018.867

**Published:** 2018-04-01

**Authors:** Sylvia Romanowska, Glenda MacQueen, Benjamin I Goldstein, JianLi Wang, Sidney H Kennedy, Signe Bray, Catherine Lebel, Jean Addington

**Affiliations:** 1 Hotchkiss Brain Institute, University of Calgary; 2 University of Toronto; 3 Institute of Mental Health Research, University of Ottawa; 4 University Health Network, Toronto

## Abstract

**Background:**

Neurocognitive deficits are associated with many serious mental illnesses (SMI), including schizophrenia, bipolar disorder, and major depressive disorder, and have been found to negatively impact social and occupational outcomes, clinical prognosis, and overall quality of life. These deficits have also been observed in people in earlier phases of schizophrenia, specifically in young people at clinical high risk (CHR) of psychosis. In these youth, neurocognitive deficits present at a level intermediate to healthy controls and those with early psychosis, indicating that mild impairments in neurocognitive functioning may be early markers of illness development. It is possible that neurocognitive deficits may be present in young people at risk of a range of SMI beyond the psychosis-spectrum, including affective and anxiety disorders. The aim of this study was to compare neurocognitive functioning in a sample of youth at risk of SMI across the different clinical stages described by McGorry and colleagues and compare them to healthy controls (HCs). It was hypothesized that participants in the later stages of risk, characterized by the presence of subthreshold psychiatric symptoms or attenuated syndromes, would exhibit impairments in neurocognitive performance compared to HCs and asymptomatic youth at familial high risk.

**Methods:**

This was an observational, cross-sectional study of 243 male and female individuals between the ages of 12–26. The sample consists of participants in the Canadian Psychiatric Risk and Outcome Study (PROCAN) and included: asymptomatic participants at familial high risk for SMI (Stage 0; n=41); youth with early mood or anxiety symptoms (Stage 1a; n=52); youth with attenuated psychotic or affective syndromes and distress (Stages 1b; n=108); and HCs (n=42). The neurocognitive battery included the WRAT-4 reading task, WASI Vocabulary and WASI Matrix Reasoning tasks, and the Measurement and Treatment Research to Improve Cognition in Schizophrenia (MATRICS) Consensus Cognitive Battery (MCCB). All neurocognitive tasks were administered at baseline. Group differences in neurocognitive performance were analyzed using MANCOVA/ANCOVA analyses. Covariates included age and sex.

**Results:**

Subjects in Stage 0 and Stage 1a did not significantly differ from any group. Subjects in Stage 1b (attenuated syndromes) had significantly lower neurocognitive scores in the domains of speed of processing, working memory, attention/vigilance and reasoning and problem solving, and on composite scores of neurocognitive performance and full-scale IQ compared to HCs. A secondary analysis demonstrated that subjects in Stage 1b who met CHR status according to Criteria of Psychosis-risk Syndromes (n=83) had lower scores in the domains of working memory, verbal learning, and reasoning and problem solving and on the overall composite score than the other participants in Stage 1b who did not meet CHR criteria.

**Discussion:**

This study provides evidence for a growing literature which suggests that neurocognitive deficits may be markers of susceptibility for SMI development. It also increases what is known about neurocognitive performance associated with different stages of risk for SMI. Identification of such impairments could aid with detection of early mental health problems prior to illness onset.

